# Estimating intra-seasonal photosynthetic discrimination and water use efficiency using δ^13^C of leaf sucrose in Scots pine

**DOI:** 10.1093/jxb/erac413

**Published:** 2022-10-18

**Authors:** Yu Tang, Paulina Schiestl-Aalto, Marco M Lehmann, Matthias Saurer, Elina Sahlstedt, Pasi Kolari, Kersti Leppä, Jaana Bäck, Katja T Rinne-Garmston

**Affiliations:** Bioeconomy and Environment Unit, Natural Resources Institute Finland (Luke), Latokartanonkaari 9, 00790, Helsinki, Finland; Institute for Atmospheric and Earth System Research (INAR)/Forest Sciences, Faculty of Agriculture and Forestry, University of Helsinki, P.O. Box 27, 00014, Helsinki, Finland; Institute for Atmospheric and Earth System Research (INAR)/Physics, Faculty of Science, University of Helsinki, P.O. Box 68, 00014, Helsinki, Finland; Forest Dynamics, Swiss Federal Institute for Forest, Snow and Landscape Research (WSL), Zürcherstrasse 111, 8903, Birmensdorf, Switzerland; Forest Dynamics, Swiss Federal Institute for Forest, Snow and Landscape Research (WSL), Zürcherstrasse 111, 8903, Birmensdorf, Switzerland; Bioeconomy and Environment Unit, Natural Resources Institute Finland (Luke), Latokartanonkaari 9, 00790, Helsinki, Finland; Institute for Atmospheric and Earth System Research (INAR)/Physics, Faculty of Science, University of Helsinki, P.O. Box 68, 00014, Helsinki, Finland; Bioeconomy and Environment Unit, Natural Resources Institute Finland (Luke), Latokartanonkaari 9, 00790, Helsinki, Finland; Institute for Atmospheric and Earth System Research (INAR)/Forest Sciences, Faculty of Agriculture and Forestry, University of Helsinki, P.O. Box 27, 00014, Helsinki, Finland; Bioeconomy and Environment Unit, Natural Resources Institute Finland (Luke), Latokartanonkaari 9, 00790, Helsinki, Finland; MPI of Molecular Plant Physiology, Germany

**Keywords:** Cavity-ringdown spectrophotometer, photosynthetic carbon isotope discrimination model, *Pinus sylvestris* L, starch, total organic matter (TOM), water-soluble carbohydrates (WSC)

## Abstract

Sucrose has a unique role in recording environmental and physiological signals during photosynthesis in its carbon isotope composition (δ^13^C) and transport of the signal to tree rings. Yet, instead of sucrose, total organic matter (TOM) or water-soluble carbohydrates (WSC) are typically analysed in studies that follow δ^13^C signals within trees. To study how the choice of organic material may bias the interpretation of δ^13^C records, we used mature field-grown Scots pine (*Pinus sylvestris*) to compare for the first time δ^13^C of different leaf carbon pools with δ^13^C of assimilates estimated by a chamber-Picarro system (δ^13^C_A_Picarro_), and a photosynthetic discrimination model (δ^13^C_A_model_). Compared with sucrose, the other tested carbon pools, such as TOM and WSC, poorly recorded the seasonal trends or absolute values of δ^13^C_A_Picarro_ and δ^13^C_A_model_. Consequently, in comparison with the other carbon pools, sucrose δ^13^C was superior for reconstructing changes in intrinsic water use efficiency (iWUE), agreeing in both absolute values and intra-seasonal variations with iWUE estimated from gas exchange. Thus, deriving iWUE and environmental signals from δ^13^C of bulk organic matter can lead to misinterpretation. Our findings underscore the advantage of using sucrose δ^13^C to understand plant physiological responses in depth.

## Teaser

Comparison of assimilate δ^13^C values estimated from different leaf carbon pools, Picarro measurements, and modelled data demonstrates the potential for misinterpretation arising from bulk organic matter δ^13^C analysis.

## Introduction

Stable carbon isotope composition (δ^13^C) of leaves and tree rings has been widely used to reconstruct past environmental and physiological signals, such as temperature (Young *et al.*, 2019), radiation (Helama *et al.*, 2018), humidity (Liu *et al.*, 2018), and intrinsic water use efficiency (iWUE) (Bauters *et al.*, 2020). These reconstructions rely upon sensitive response of δ^13^C of new assimilates (δ^13^C_A_) to environmental changes (McCarroll and Loader, 2004), and preservation of this environment-driven δ^13^C signal in tree rings (Rinne *et al.*, 2015b). As an important photosynthetic product and the predominant transport sugar (Rennie and Turgeon, 2009; Julius *et al.*, 2017), sucrose plays a unique role in recording the δ^13^C signal within trees. Rather than sucrose, however, water-soluble carbohydrates (WSC, which include sugars and sugar alcohols), water-soluble organic matter (which includes amino acids, organic acids, and phenolic compounds in addition to WSC), and total organic matter (TOM) are typically analysed in studies that seek to understand the recording and transporting of the δ^13^C signal in trees at intra-seasonal scale (e.g. Gessler *et al.*, 2009a; Offermann *et al.*, 2011). The isotopic analysis of bulk organic matter may bias our understanding of how the δ^13^C signal is archived in tree tissues, considering that many components included in bulk matter differ in their δ^13^C values compared with new assimilates due to fractionations during secondary metabolism (Brugnoli and Farquhar, 2000; Bowling *et al.*, 2008). There are further concerns that a high and changing proportion of previously formed compounds in bulk carbon pools may outweigh the influence of new assimilates, which would result in blurred intra-seasonal environmental and physiological signals in δ^13^C of bulk organic matter.

δ^13^C_A_ is more ^13^C-depleted than δ^13^C of atmospheric CO_2_ because of a series of fractionation processes during photosynthesis, impacted by stomatal conductance (Farquhar *et al.*, 1982), mesophyll conductance (*g*_m_) (Schiestl-Aalto *et al.*, 2021), Rubisco activity (Farquhar *et al.*, 1982), photorespiration (Tcherkez, 2006), and mitochondrial respiration (Ghashghaie *et al.*, 2003). With knowledge of these fractionation processes, it is possible to estimate δ^13^C_A_, for example using the most widely used steady-state model, developed by Farquhar *et al.* (1989), and its modified versions (Wingate *et al.*, 2007; Seibt *et al.*, 2008; Busch *et al.*, 2020). However, uncertainties exist for modeled δ^13^C_A_ values due to limited understanding of fractionations associated with *g*_m_, mitochondrial respiration, and photorespiration (Busch *et al.*, 2020). δ^13^C_A_ can also be derived from δ^13^C of the CO_2_ flux entering the leaf (Farquhar *et al.*, 1989), using leaf gas exchange chambers connected to optical spectrometers (Wingate *et al.*, 2010; Stangl *et al.*, 2019; Schiestl-Aalto *et al.*, 2021). Although chamber measurements can provide data at high temporal resolution, they have limitations in terms of high measurement noise (Cernusak, 2020). One approach to evaluate the reliability and accuracy of chamber-derived and modeled δ^13^C_A_ values is to compare them with δ^13^C of leaf sucrose, the main end product of photosynthesis. Even though leaf sucrose may also carry some information on use of reserves, which have different δ^13^C values from recent photosynthates (Bowling *et al.*, 2008), it can be expected to have δ^13^C values closer to primary photosynthates compared with bulk matter (Rinne *et al.*, 2015a). Yet, we are not aware of studies that have compared δ^13^C of leaf sucrose with chamber-derived or modeled δ^13^C_A_ data at intra-seasonal scale.

Since δ^13^C_A_ is mediated by the interplay of photosynthetic rate and stomatal conductance (McCarroll and Loader, 2004), the ratio of these two variables, i.e. iWUE, can be derived from δ^13^C_A_. However, the accuracy of iWUE estimates from δ^13^C of leaf bulk organic matter on intra-seasonal scale has sometimes been questioned (Tarin *et al.*, 2020), probably because of the deviation of the bulk organic matter δ^13^C signal from δ^13^C_A_. In comparison, iWUE derived from δ^13^C of phloem exudates, which mainly consist of sucrose (Gessler *et al.*, 2004), agreed well with iWUE calculated from gas exchange measurements in the study of Gimeno *et al.* (2021). Likewise, Merchant *et al.* (2011) found that, in comparison with δ^13^C of leaf soluble carbon, δ^13^C of sucrose from both leaves and phloem sap was more tightly coupled to the ratio of intercellular to ambient CO_2_ concentrations (*c*_i_/*c*_a_), a parameter closely linked to iWUE. This indicates that δ^13^C of sucrose has an advantage over δ^13^C of bulk organic matter in understanding how the iWUE signal is formed in leaves.

Another reason for the deviation of δ^13^C of leaf carbon pools from δ^13^C_A_ is that the former reflects a time-integrated signal. Streit *et al.* (2012) found that the mean residence times of the ^13^C labels in leaf sucrose and WSC of mature *Larix decidua* after ^13^CO_2_ pulse labelling were 2 and 4 d, respectively, consistent with the report of residence times from 1 to 5 d for leaf WSC in mature *Pinus pinaster* (Desalme *et al.*, 2017). Similarly, Leppä *et al.* (2022) suggested that the leaf sugar pool was a composite of sugars formed between 2 to over 5 d, by assuming a constant size of leaf sugar pool with well-mixed new and old assimilates in a photosynthetic isotope discrimination model. These pieces of evidence demonstrate that different carbon pools integrate δ^13^C_A_ and thereby environmental and physiological signals across varying time spans, depending on their turnover rates. Such mixing effect or carry-over effect of leaf assimilates is still poorly understood but should be carefully considered when explaining the intra-seasonal changes in δ^13^C of leaf carbon pools.

In the current study, we investigate how the choice of material for δ^13^C analysis impacts our understanding of the recording of environment-driven δ^13^C signal in leaves. We have three specific aims: (i) to compare three different approaches for estimating intra-seasonal δ^13^C_A_, as derived from different leaf carbon pools, from gas exchange measurements connected to a Picarro (δ^13^C_A_Picarro_), and from a photosynthetic isotope discrimination model (δ^13^C_A_model_); (ii) to study how environmental signals, including photosynthetically active radiation (PAR), air temperature (*T*), relative humidity (RH), and vapor pressure deficit (VPD), were recorded in δ^13^C of different carbon pools, including sucrose, pinitol, starch, WSC, and TOM; and (iii) to evaluate the accuracy of iWUE estimated from the δ^13^C of different carbon pools (iWUE_iso_), with and without incorporating *g*_m_, which was done by comparing the estimates with iWUE derived from gas exchange data (iWUE_gas_).

## Materials and methods

### Site description

The study site Hyytiälä SMEAR II is a boreal forest located in southern Finland (61°51ʹN, 24°17ʹE, 170 m a.s.l.). Being slash-burned and sown in 1962, the site was dominated by 56-year-old Scots pine (*Pinus sylvestris* L.) trees in 2018. Other tree species include Norway spruce (*Picea abies* L. Karst) and birch (*Betula* spp.). The stand density for all trees taller than 1.3 m was 1177 ha^−1^, and the dominant height was 18 m in summer 2016 (Schiestl-Aalto *et al.*, 2019). The soil is a haplic podzol on glacial till (FAO–UNESCO, 1990), with a mineral soil layer depth of 0.5–0.7 m over the bedrock (Schiestl-Aalto *et al.*, 2019). During 1981–2010, mean annual *T* was +3.5 °C with mean monthly *T* varying from –7.7 °C in February to 16.0 °C in July; and mean annual precipitation was 711 mm, almost evenly distributed throughout the year (Pirinen *et al.*, 2012).

### Environmental and growth data

Environmental data for the study site were obtained from the AVAA Smart SMEAR portal (https://smear.avaa.csc.fi/). Volumetric soil moisture (m^3^ m^−3^) in the A horizon was monitored 20–40 times per day by Campbell TDR100 time-domain reflectometers at five locations. Precipitation (mm) accumulated at 1 min intervals was recorded by a Vaisala FD12P weather sensor. PAR (µmol m^−2^ s^−1^) in the wavelength range 400–700 nm at 35 m height was captured every minute by a LI-COR (Lincoln, NE, USA) Li-190SZ quantum sensor. Air pressure at ground level, *P*_amb_ (kPa), was measured every minute by a Druck DPI 260 barometer. *T* (°C) at 16.8 m height was measured every minute with a Pt100 temperature sensor inside ventilated custom-made radiation shield. RH (%) at 16.8 m height was recorded every minute by a Rotronic MP102H RH sensor. For each air *T* and RH measurement, VPD in kPa was calculated according to Eqs 1, 2:


$$\text{VPD}=e_{\text{s}}-e_{\text{a}}=e_{\text{s}}\times (100-\text{RH})/100$$ (1)

$$e_{\text{s}}=610.7\times 10^{\frac{7.5T}{237.3+T}}/1000,$$ (2)

where *e*_s_ is saturated vapor pressure of the air (kPa) when *T* is given in °C, and *e*_a_ is the vapor pressure in the air (kPa). Daytime means of PAR, *T*, RH, VPD, soil moisture, and daily accumulative precipitation were calculated defining daytime as the period 2 h after sunrise to 2 h before sunset.

Needle growth was traced by measuring length increment of needles from 15 shoots at top or middle canopy of three mature Scots pine trees. Measurements were done two or three times a week from May to August until the full expansion of new needles. The growth period was defined as the time period when 5–95% of full length was achieved.

### Needle sampling, extraction and purification of WSC and starch

One-year-old needles (1N) and current-year needles (0N) were collected separately from five mature Scots pine trees at sun-exposed positions 2 m below the top of the canopy. The top canopy of the trees was accessible using a walk-in scaffolding tower and 10 m-long branch scissors. Needle samples were harvested between 13.00 h and 16.00 h 20 times in 2018: four or five times per month from May to August, twice in September and October. All sampling days except 4 July were non-rainy days. Collection of 0N started when their length had reached 1 cm. 0N were pooled from five trees for the first two sampling days when their length was small. After collection, samples were put in a cool box, micro-waved as soon as possible at 600 W for 1 min to stop enzymatic and metabolic activities (Wanek *et al.*, 2001), dried for 24 h at 60 °C in an oven and homogenized into a fine powder using FastPrep-24 (MP Biomedicals, Irvine, CA, USA).

Extraction and purification of needle WSC were performed according to Wanek *et al.* (2001) and Rinne *et al.* (2012). In brief, 60 mg of needle powder was transferred into a 2 ml reaction vial and re-suspended in 1.5 ml of deionized water. The vials were placed in a water bath at 85 °C for 30 min, cooled for 30 min, and centrifuged at 10 000 *g* for 2 min. The separated supernatant was then purified by three types of sample preparation cartridges (Dionex OnGuard II H, A, and P cartridges, Thermo Fisher Scientific, Waltham, MA, USA) to remove amino acids, organic acids, and phenolic compounds. The purified WSC samples were subsequently freeze-dried, dissolved in 1 ml deionized water, and filtered through a 0.45 μm syringe filter and stored at –20 °C until isotope analysis.

Starch was extracted from the pellet of the hot water extraction by enzymatic hydrolysis (Wanek *et al.*, 2001; Lehmann *et al.*, 2019). The pellet in each reaction vial was washed with 1.2 ml methanol–chloroform–water (12:5:3, v/v/v) solution four times and with 1.2 ml deionized water three times to remove lipids. Lipid-free pellet in each vial was re-suspended in 0.75 ml deionized water and boiled at 99 °C for 15 min in a water bath to gelatinize the starch. Starch in the pellet was then hydrolysed at 85 °C in a water bath for 2 h after adding 0.25 ml purified (by Vivaspin 15R, Sartorius, Göttingen, Germany) α-amylase (EC 3.2.1.1, Sigma-Aldrich, Buchs, Switzerland) solution of 3000 U ml^−1^. The hydrolysed starch was later separated from enzymatic residues with centrifugation filters (Vivaspin 500, Sartorius) and stored at –20 °C until isotope analysis. An identical treatment principle (Werner and Brand, 2001) was applied to two maize starch standards (Fluka, Buchs, Switzerland), two wheat starch standards (Fluka, Buchs) and four blanks with every batch of 40 samples.

### δ^13^C values of leaf carbon pools

δ^13^C values of TOM, WSC, and starch (δ^13^C_TOM_, δ^13^C_WSC_, and δ^13^C_starch_, respectively) were measured at the Stable Isotope Laboratory of Luke (‘SILL’, Natural Resources Institute, Finland), using an elemental analyser (Europa EA-GSL, Sercon Ltd, Crewe, UK) coupled to an isotope ratio mass spectrometry (20-22 IRMS, Sercon Ltd). Milled needle material was weighed into tin capsules (5 × 9 mm, Säntis, Teufen, Switzerland). Aliquots of purified WSC, hydrolysed starch, as well as standards and blanks were pipetted into the tin capsules, freeze-dried, and wrapped. The δ^13^C values of the samples were reported as a ‰ difference from the international Vienna-Pee Dee Belemnite (V-PDB) standard:


$$\;\delta {\;}^{13}\text{C}=\left(\frac{R_{\text{sample}}}{R_{\text{standard}}}-1\right)\times 1000$$ (3)

where *R*_sample_ and *R*_standard_ are the ^13^C/^12^C ratio in a sample and standard, respectively. δ^13^C results were calibrated against IAEA-CH3 (cellulose, –24.724‰), IAEA-CH7 (polyethylene, –32.151‰), and in-house (sucrose, –12.22‰) reference material. Measurement precision determined from multiple analyses of a quality control material was 0.1‰ (SD).

Compound-specific isotope analysis (CSIA) was done at the Stable Isotope Research Laboratory of WSL (Birmensdorf, Switzerland) using a high-performance liquid chromatography (HPLC)-IRMS system with a Thermo LC Isolink interface (Krummen *et al.*, 2004; Rinne *et al.*, 2012). Four sugars and sugar alcohols with a concentration of 20–180 ng C μl^−1^ were detected for HPLC-IRMS δ^13^C analysis: sucrose, glucose, fructose, and pinitol/*myo*-inositol. As pinitol and *myo*-inositol co-elute from the analytical column and the two compounds have a close relation in biosynthetic pathways and in stress-related processes (Rinne *et al.*, 2012 and references therein), they were treated as one compound (referred to as ‘pinitol’ hereafter). A series of compound-matched external standard solutions with a range of concentrations of 20, 40, 60, 90, 120, and 180 ng C μl^−1^ was analysed between every 10 samples. The compound-specific δ^13^C results of samples were corrected by the linearity of the peak area to the δ^13^C values of the standards (Rinne *et al.*, 2012). δ^13^C values of sucrose and pinitol (δ^13^C_sucrose_ and δ^13^C_pinitol_, respectively) were reported in the current study with measurement precision of 0.24‰ (SD) and 0.19‰ (SD), respectively.

### Online chamber-Picarro measurement

Shoot gas exchange measurements were performed with automated chamber systems, consisting of chambers, sample tubing, and gas analysers. Two non-airtight shoot chambers made of transparent acrylic plastic were installed at the uppermost canopy of one sampling tree. One chamber with 1 dm^3^ volume enclosed a 1-year-old shoot with all the needles gently bent to form a plane (Supplementary Fig. S1); the other with 2.1 dm^3^ volume enabled the insert of a free-shape 1-year-old shoot (Supplementary Fig. S1). Both inserted shoots were debudded to prevent new growth. Chambers were attached to the same measuring shoots over the whole growing season, i.e. not swapping to different shoots, to avoid any possible disturbance to the measuring shoots. The shoot chambers were equipped with a fan and intermittently closed one by one for 65s 50–80 times per day. During the chamber closure, the sample air was drawn along separate polytetrafluoroethylene tubes (internal diameter 4 mm, length 73 m) to the gas analysers (G2201-I, Picarro, Santa Clara, CA, USA; and LI-840, LI-COR) and the sample air flow was compensated by ambient air leaking freely into the shoot chambers.

Both ^12^CO_2_ and ^13^CO_2_ fluxes were determined by fitting non-linear regression to the concentrations of ^12^CO_2_ and ^13^CO_2_ at 0.5 s intervals captured by the Picarro during the first 5–50 s of chamber closure (Kolari *et al.*, 2012). ^12^CO_2_ and ^13^CO_2_ concentrations were calibrated against reference CO_2_ gases (Air Liquide, Houston, TX, USA) with CO_2_ concentrations of 400 ppm and δ^13^C of –19‰ and –3.1‰. Both ^12^CO_2_ and ^13^CO_2_ fluxes were calculated for all-sided needle area data (Kolari *et al.*, 2007). δ^13^C_A_Picarro_ was calculated from the ratio of ^13^CO_2_ flux to ^12^CO_2_ flux in comparison with the V-PDB standard (Eq. 3). The calculation procedure and equations are described in detail in Kolari *et al.* (2012). Raw δ^13^C_A_Picarro_ data were discarded when CO_2_ flux was lower than 0.5 μmol m^−2^ s^−1^, as a low CO_2_ flux rate decreases the precision of the calculated δ^13^C data. Flux weighted daytime mean δ^13^C_A_Picarro_ for each chamber was calculated.

CO_2_ and H_2_O concentrations were also recorded by the LI-840 at 5 s intervals. The fluxes of CO_2_ and H_2_O were calculated according to non-linear regression fitted to changes of concentrations from 5 to 35 s after chamber closure (Kolari *et al.*, 2012). CO_2_ concentrations were calibrated against control gases (Sundbyberg, Sweden) with CO_2_ concentrations of 303 and 400 ppm. H_2_O flux data were omitted when RH exceeded 75%, as water is adsorbed on the chamber walls at high humidity, which makes transpiration measurement unreliable (Altimir *et al.*, 2006).

### Photosynthetic isotope discrimination model

δ^13^C_A_model_ was estimated by the classic photosynthetic carbon isotope discrimination model (Farquhar *et al.*, 1982), which describes carbon isotope fractionations due to stomatal conductance, *g*_m_, carboxylation, mitochondrial respiration, and photorespiration (Eq. 4).


$$\Delta =a+(b-a)\frac{c_{i}}{c_{\text{a}}}-(b-a_{\text{m}})\frac{A}{g_{\text{m}}c_{\text{a}}}-f\frac{\Gamma^{*}}{c_{\text{a}}}-\frac{R_{\text{d}}}{A+R_{\text{d}}}e\frac{c_{i}-\Gamma^{*}}{c_{\text{a}}}$$ (4)

where Δ is photosynthetic discrimination; *a* is the fractionation due to gaseous diffusion of CO_2_ through stomata, 4.4‰; *b* is the fractionation due to carboxylation, 29‰; *a*_m_ is the fractionation during the mesophyll CO_2_ transfer, 1.8‰; *c*_i_ is calculated from Eq. 5; *A* is CO_2_ flux in μmol m^−2^ s^−1^; *g*_m_ is 0.127 mol m^−2^ s^−1^ for Scots pine (corrected from 0.33 mol m^−2^ s^−1^ for all-sided needle area; Stangl *et al.*, 2019); *f* is the fractionation during photorespiration, 8‰ (Ghashghaie *et al.*, 2003); Γ^*^ is the CO_2_ compensation point in the absence of dark respiration in μmol mol^−1^, estimated according to Eq. 6 (Bernacchi *et al.*, 2001); *e* is the fractionation during day respiration, –6‰ (Ghashghaie *et al.*, 2003); and *R*_d_ is the mitochondrial respiration estimated from Eq. 7 (Bernacchi *et al.*, 2001).


$$c_{\text{i}}=c_{\text{a}}-1.6\cdot {\text{iWUE}}_{\text{gas}}$$ (5)

$$\Gamma_{*}=42.75\cdot \text{exp}\{37830\cdot (T_{l}-25)/[298\cdot R\cdot (T_{l}+273.15)]\}$$ (6)

$$R_{\text{d}}=R_{\text{d0}}\cdot \text{exp}\{H_{\alpha }\cdot (T_{\text{l}}-25)/[298\cdot R\cdot (T_{\text{l}}+273.15)]\}$$ (7)

where *T*_l_ is leaf temperature in °C, taken as air *T* measured inside the chamber; *R* is the universal gas constant (8.3145 J mol^−1^ K^−1^); *R*_d0_ and *H*_α_ are parameters predicted by fitting Eq. 7 to the night-time chamber flux data. iWUE_gas_ in ppm was calculated according to Eqs 8, 9.


$${\text{iWUE}}_{\text{gas}}=A/g_{\text{s}}$$ (8)

$$E=g_{\text{s}}\cdot (e_{\text{s}}-e_{\text{a}})/P_{\text{amb}}$$ (9)

where *g*_s_ is stomatal conductance in mmol m^−2^ s^−1^ and *E* is the H_2_O flux in mmol m^−2^ s^−1^.

Equation 4 assumes the substrate for *R*_d_ is new assimilates, which Wingate *et al.* (2007) have shown to fit observations well during daytime when *A* + *R*_d_ dominates over *R*_d_. Eq. 7 was fitted to night-time chamber flux data and thus assumes *R*_d_ is not light inhibited during daytime. We evaluated the impact of light-inhibited *R*_d_ on δ^13^C_A_model_ by setting *R*_d_=0 in Eq. 4. Due to a negligible impact of light-inhibited *R*_d_ (Supplementary Fig. S2) and a better fit to chamber-derived δ^13^C_A_ with non-light-inhibited *R*_d_ reported by Wingate *et al.* (2007), we reported the δ^13^C_A_model_ results with non-light-inhibited *R*_d_. In addition, the choice of *f* value had limited impact on δ^13^C_A_model_ results, with *f*=11‰ (Tcherkez, 2006) giving 0.2‰ higher δ^13^C_A_model_ results on average, compared with *f*=8‰ (Ghashghaie *et al.*, 2003). We reported results with *f*=8‰ due to a better fit with chamber-derived δ^13^C_A_Picarro_.

With modeled Δ values from Eq. 4 and measured δ^13^C of ambient air (δ^13^C_air_), δ^13^C_A_ can be modeled from Eq. 10:


$$\;\delta {\;}^{13}C_{\text{A}}=1000\times (\;\delta {\;}^{13}{\text{C}}_{\text{air}}-\Delta )/(\Delta +1000)$$ (10)

Input environmental data of the model, including *A*, *E*, RH, *T*_l_, *c*_a_, and δ^13^C_air_, were obtained from gas exchange measurements. Flux weighted daytime mean δ^13^C_A_model_ was calculated.

### iWUE estimations

iWUE was estimated in three ways: (i) from gas exchange data (iWUE_gas_) (Eqs. 8, 9), (ii) from δ^13^C of different carbon pools via a complex iWUE model (iWUE_iso_) (Eq. 11), and (iii) from δ^13^C of different carbon pools via a simple iWUE model without consideration of *g*_m_, mitochondrial respiration, and photorespiration (iWUE_iso_’) (Eq. 12).


$${\text{iWUE}}_{\text{iso}}=\frac{c_{\text{a}}}{1.6}\times \frac{b-\Delta -f\frac{\Gamma^{*}}{c_{\text{a}}}-(b-a_{\text{m}})\frac{A}{g_{\text{m}}c_{\text{a}}}+\frac{R_{\text{d}}}{A+R_{\text{d}}}e\frac{\Gamma^{*}}{c_{\text{a}}}}{b-a-\frac{R_{\text{d}}}{A+R_{\text{d}}}e}$$ (11)

$${\text{iWUE}}_{\text{iso}}{}^{\prime }=c_{\text{a}}\times (b^{\prime }-\Delta )/[1.6\times (b^{\prime }-a)]$$ (12)

Δ is calculated from Eq. 10 using δ^13^C of different carbon pools as input of δ^13^C_A_. *bʹ* in Eq. 12 is the net discrimination due to carboxylation, 27‰. Daytime means of gas exchange data, including δ^13^C_air_, *T*_l_, *c*_a_, and *A*, were measured by the Picarro and used as input data. δ^13^C_air_ and *c*_a_ were measured before chamber closure. iWUE_iso_ and iWUE_iso_*ʹ* were compared with iWUE_gas_ integrated over previous days (from 0–12 d) to account for possible carry-over effects (Eq. 13).

### Data analysis

We applied a linear mixed-effects model to examine (i) the difference between δ^13^C_A_Picarro_ and δ^13^C_A_model_ (Supplementary Table S1), (ii) the δ^13^C difference between 1N and 0N for different carbon pools (Supplementary Table S2), and (iii) the temporal trends in the δ^13^C series during a certain period (Supplementary Table S3). Tree identifier (1, 2, 3, 4, 5) or chamber identifier (1, 2) was used as a random term. A full model was tested with the effect of day of year and generation (1N, 0N) or method (from the chamber-Picarro system or from the isotope discrimination model) and their interactions. If not significantly different from the full model, a reduced model without the interaction was applied. The linear mixed-effects modeling was performed in R with the R package ‘nlme’ (Pinheiro *et al.*, 2022).

We calculated Spearman’s correlation between δ^13^C of different leaf carbon pools and δ^13^C_A_Picarro_ and δ^13^C_A_model_ (aim (i)), Spearman’s correlation between δ^13^C of different carbon pools and PAR, RH, *T*, and VPD (aim (ii)), and Spearman’s correlation between δ^13^C of different carbon pools and iWUE_gas_ (aim (iii)). Means of the δ^13^C series from five sampling trees or from two chamber systems were used in correlation analysis. All variables, except δ^13^C of different leaf carbon pools, were integrated over previous day signals with varying weights, according to Eq. 13.


$$X_{t}^{*}=\left(\underset{i=0}{\sum^{n}}\;\lambda {\;}^{i}\times X_{t-i}\right)/\underset{i=0}{\sum^{n}}\;\lambda {\;}^{i}$$ (13)

where $X_{t}^{*}$ is the integrated variable on day *t*; *i* is the number of days prior to day *t*; *n* is the number of days calculated, which varies from 0 to 12 d at an interval of 1 d; λ is the previous day weight, which varies from 0.1 to 1 at an interval of 0.1; *X*_*t*−*i*_ is the variable at day *t*−*i*. λ defines the percentage of leaf carbon pools in the current day that is reserved in the following day. Using Eq. 13, we investigated the carry-over effect resulting from the possible presence of sugars and other compounds in the sample originating from the days preceding the actual sampling date. All statistical analyses were done in R version 4.0.0 (R Core Team, 2020).

## Results

### Environmental conditions

**Fig. 1.**
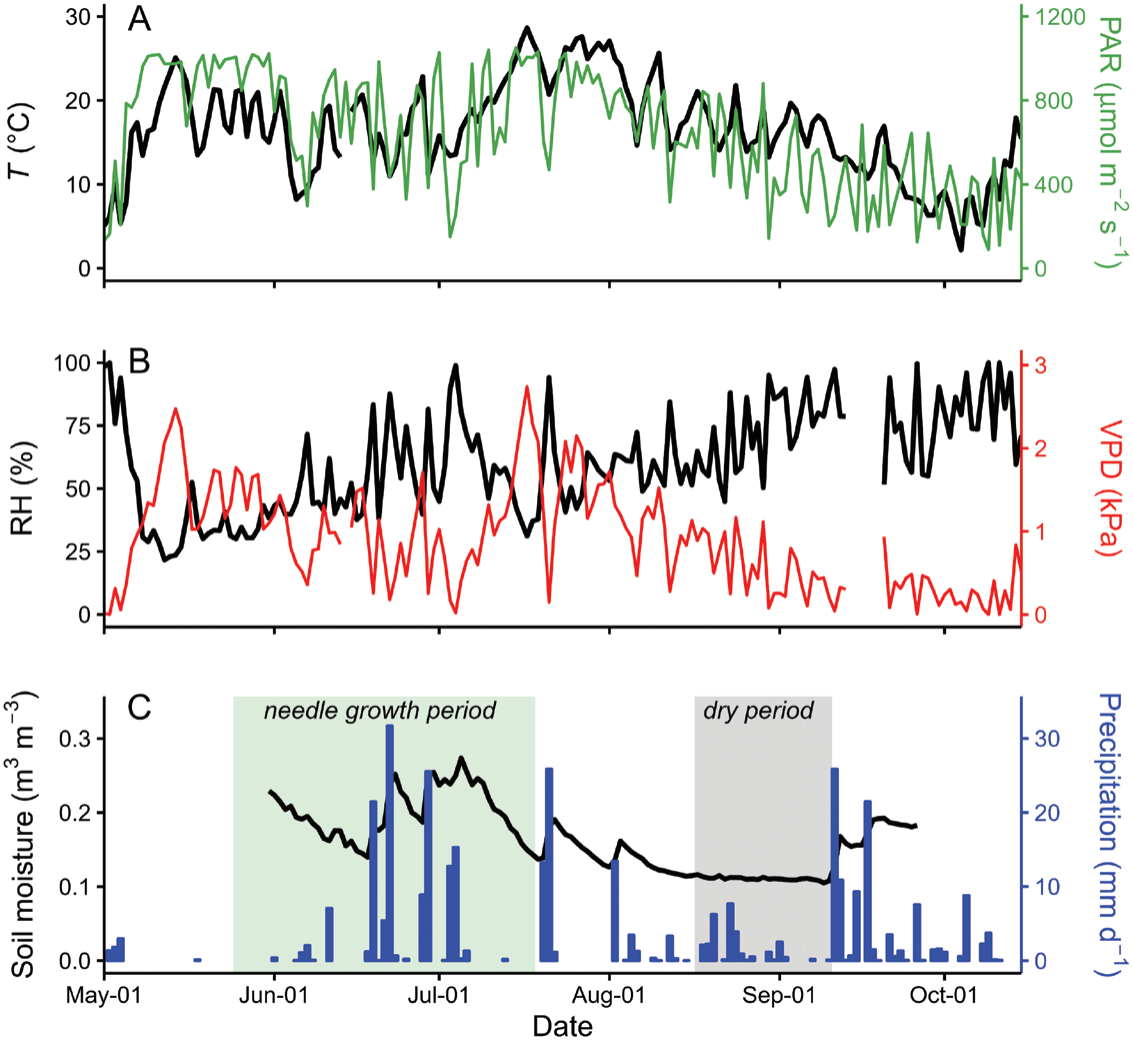
Seasonal course of environmental conditions at Hyytiälä during the growing season of 2018. (A) Daytime mean air temperature (*T*) and photosynthetically active radiation (PAR), (B) daytime mean vapor pressure deficit (VPD) and relative humidity (RH), and (C) daily accumulated precipitation and daytime mean soil moisture in topsoil. The needle growth period is shaded in green, and the dry period with soil moisture around 0.1 m^3^ m^−3^ in gray.

Daytime mean PAR and *T* exhibited a simultaneous sharp increase in the beginning of May (Fig. 1A). From then onwards, PAR values remained high until mid-July, but showed large day-to-day variations from June onwards, coinciding with days with high precipitation (Fig. 1A, C). *T*, on the other hand, reached maximum values (>25 °C) in the second half of July (Fig. 1A). From late July till the end of the growing season, both PAR and *T* displayed a gradual decreasing trend. During the studied period, VPD almost mirrored the changes in *T* (Pearson’s *r*=0.74, *P*<0.001; Fig. 1A, B), whereas RH was negatively correlated with *T* (Pearson’s *r*=–0.39, *P*<0.001; Fig. 1A, B).

The site experienced a dry period in the later part of the growing season, lasting from 16 August to 10 September (Fig. 1C). During the dry period, soil moisture in the A horizon dropped close to the wilting point of 0.1 m^3^ m^−3^ (Mecke *et al.*, 2002), and soil water potential in the A horizon was below –0.5 MPa.

### Comparing δ^13^C values

#### Chamber-derived and modeled δ^13^C_A_

**Fig. 2.**
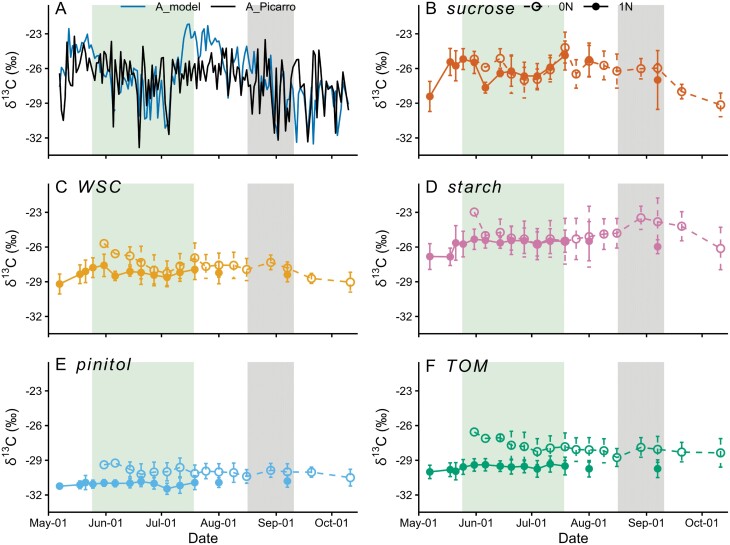
δ^13^C of assimilates and different carbon pools in current-year needles (0N) and 1-year-old needles (1N) of Scots pine at Hyytiälä during the growing season of 2018. (A) δ^13^C of assimilates estimated by the chamber-Picarro system (A_Picarro) and by the photosynthetic isotope discrimination model (A_model), (B) δ^13^C of sucrose, (C) δ^13^C of water-soluble carbohydrates (WSC), (D) δ^13^C of starch, (E) δ^13^C of pinitol, and (F) δ^13^C of total organic matter (TOM). The needle growth period is shaded in green, and the dry period in gray. Error bars represent SD of five trees.

δ^13^C_A_Picarro_ and δ^13^C_A_model_ shared a similar seasonal pattern, with an increasing trend in the first half of May, a gradual decline from mid-May to early July, and a declining trend from August until the end of the growing season (Fig. 2A). Over the growing season, the two datasets had a significant positive correlation (Pearson’s *r*=0.43, *P*<0.001). Overall, there was no significant difference (Supplementary Table S1) between δ^13^C_A_model_ (–26.6 ± 2.5‰) and δ^13^C_A_Picarro_ (–26.9 ± 2.0‰). However, between 11 July and 2 August, a period marked by the highest daytime *T* (25.0 ± 2.4 °C, Fig. 1A) and the highest global radiation (414 ± 76 W m^−2^), δ^13^C_A_model_ was on average 2.7‰ higher than δ^13^C_A_Picarro_ (Supplementary Table S1).

#### δ^13^C of leaf carbon pools of two needle generations

δ^13^C_sucrose_ did not differ between the needle generations after the first three sampling days of 0N (Fig. 2B; Supplementary Table S2). By contrast, δ^13^C_WSC_ was clearly higher in 0N than in 1N, but with a rather stable δ^13^C offset (0.6 ± 0.2‰) after the first three sampling days of 0N (Fig. 2C; Supplementary Table S2). For starch, pinitol, and TOM, 0N were also more ^13^C-enriched throughout the growing season (Supplementary Table S2), but the ^13^C-enrichment was unstable, ranging from 0 to 2.2‰ (Fig. 2D), from 0.6 to 1.8‰ (Fig. 2E), and from 1.4 to 2.8‰ (Fig. 2F), respectively.

Since δ^13^C_sucrose_ was similar between 1N and 0N after the initial stages of needle growth, we combined their 1N and 0N series via a simple average (Supplementary Fig. S3A), to obtain a longer time series of δ^13^C data for correlation analysis. For δ^13^C_WSC_, the consistent offset (0.6‰) between 1N and 0N was first subtracted from the 0N data before calculating the averages. In this manner, the combined δ^13^C_WSC_ series represented 1N (Supplementary Fig. S3B) and was thus best suited for the comparison with δ^13^C_A_Picarro_ and δ^13^C_A_model_ that were derived from gas exchange measurements on 1N. However, we did not combine the 1N and 0N series for δ^13^C_starch_, δ^13^C_pinitol_, and δ^13^C_TOM_ because of the significant (Supplementary Table S2) and changing (Fig. 2D–F) δ^13^C offsets between the two needle generations. The combined series of δ^13^C_sucrose_ and δ^13^C_WSC_ were used in the following analysis.

#### δ^13^C of leaf carbon pools and chamber-derived and modeled δ^13^C_A_

**Fig. 3.**
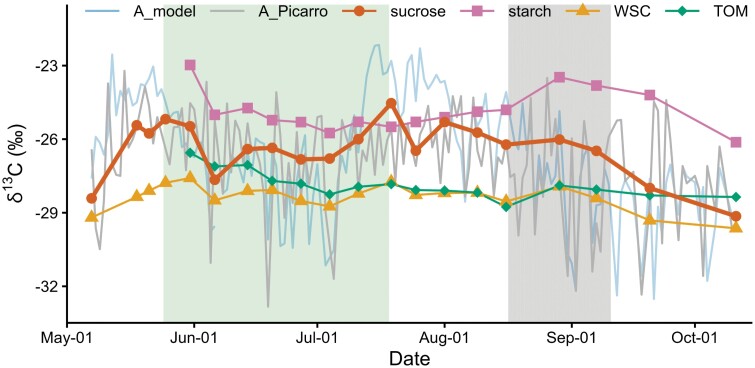
Comparison of δ^13^C of assimilates and δ^13^C of different leaf carbon pools of Scots pine at Hyytiälä during the growing season of 2018. δ^13^C of assimilates was estimated by the chamber-Picarro system (A_Picarro) and by the photosynthetic isotope discrimination model (A_model). Combined δ^13^C of sucrose (Supplementary Fig. S3A), combined δ^13^C of water-soluble carbohydrates (WSC) (Supplementary Fig. S3B), δ^13^C of starch in current-year needles (0N), and δ^13^C of total organic matter (TOM) in 0N are presented. δ^13^C of pinitol is excluded, as pinitol has not been used to estimate δ^13^C of assimilates in the literature. The needle growth period is shaded in green, and the dry period in gray.

**Fig. 4.**
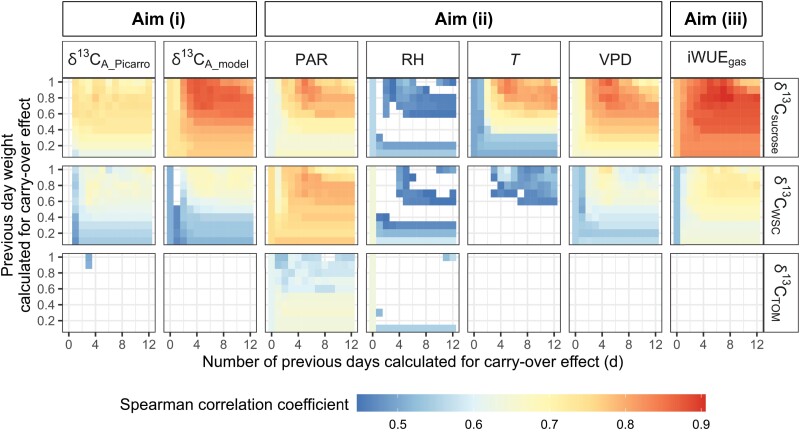
Environmental and physiological signals imprinted in δ^13^C of leaf carbon pools of Scots pine at Hyytiälä during the growing season of 2018. The analysed variables, including δ^13^C of assimilates estimated from the chamber-Picarro system (δ^13^C_A_Picarro_), δ^13^C of assimilates estimated from the photosynthetic isotope discrimination model (δ^13^C_A_model_), photosynthetically active radiation (PAR), relative humidity (RH), air temperature (*T*), vapor deficit pressure (VPD), and intrinsic water use efficiency from gas exchange data (iWUE_gas_), were integrated with a carry-over effect and a varying previous day weight (Eq. 13). Presented δ^13^C series of leaf carbon pools included combined δ^13^C of sucrose (δ^13^C_sucrose_), combined δ^13^C of water-soluble carbohydrates (δ^13^C_WSC_), and δ^13^C of total organic matter (δ^13^C_TOM_) in current-year needles. The *x*-axis represents the number of days calculated for the carry-over effect, and the *y*-axis the percentage of current-day leaf carbon pools that were reserved in the following day. Only significant results (*P<*0.05) are presented. Spearman’s correlation coefficient is indicated by the colors from blue (low values) to red (high values).

The seasonal changes and mean values of δ^13^C were similar in general for needle δ^13^C_sucrose_ (–26.4 ± 1.2‰) and the series of δ^13^C_A_Picarro_ (–26.9 ± 2.0‰) and δ^13^C_A_model_ (–26.6 ± 2.5‰) (Fig. 3). For example, all three datasets presented an inverse ‘V’ shape variation in May (Fig. 3) and a declining trend from August onwards (Supplementary Table S3). On the other hand, the declining trend in δ^13^C_A_Picarro_ and δ^13^C_A_model_ from mid-May to end of June was less evident in δ^13^C_sucrose_. This can be mainly assigned to the different temporal resolution of δ^13^C_sucrose_ series, as its individual data points aligned well with δ^13^C_A_Picarro_ and δ^13^C_A_model_ during this period. However, there were two sampling days when δ^13^C_sucrose_ clearly deviated with its relatively ^13^C-enriched value from both δ^13^C_A_Picarro_ and δ^13^C_A_model_: 4 July, a rainy day with low PAR, and 7 September, which occurred at the end of the dry period (Fig. 3). Between 11 July and 2 August, the warm period when δ^13^C_A_model_ was approximately 2.7‰ higher than δ^13^C_A_Picarro_ (Fig. 2A), δ^13^C_sucrose_ had similar values to δ^13^C_A_Picarro_, albeit somewhat more ^13^C-enriched on 19 July (Fig. 3). δ^13^C_sucrose_ had the strongest correlation with δ^13^C_A_model_ (Spearman's ρ=0.87, *P*<0.001) when a carry-over effect of 4 d and a previous day weight of 0.8 were applied (Fig. 4; Supplementary Fig. S4), and with δ^13^C_A_Picarro_ (Spearman's ρ=0.75, *P*<0.001) when a carry-over effect of 4 d and a previous day weight of 0.7 were considered (Fig. 4; Supplementary Fig. S4). However, it is worth noting that similar *r*-values were obtained in a range of combinations of previous day signal (Fig. 4).

δ^13^C_WSC_ shared a similar low-frequency trend with δ^13^C_A_Picarro_ and δ^13^C_A_model_ (Fig. 3), but the absolute values of δ^13^C_WSC_ (–28.4 ± 0.5‰) were on average 1.5‰ and 1.8‰ lower than δ^13^C_A_Picarro_ and δ^13^C_A_model_, respectively. δ^13^C_TOM_ in 0N followed the changes in and absolute values of δ^13^C_A_Picarro_ and δ^13^C_A_model_ during the initial development stages of 0N, showing a declining trend from late May to the end of June (Fig. 3; Supplementary Table S3). However, after the maturation of 0N, δ^13^C_TOM_ in 0N was almost invariant and overall lower than δ^13^C_A_Picarro_ and δ^13^C_A_model_ by 1.1‰ and 1.4‰, respectively (Fig. 3). δ^13^C_TOM_ in 1N (–29.6 ± 0.2‰) was invariant during the whole growing season, and 1.9‰ more ^13^C-depleted than its counterpart in 0N. δ^13^C_starch_ in both 1N and 0N was almost invariant from June to mid-August (Figs 2D, 3) and did not correlate with δ^13^C_A_Picarro_ or δ^13^C_A_model_. Similarly, the almost invariant δ^13^C_pinitol_ values in both 1N (–31.0 ± 0.3‰) and 0N (–30.1 ± 0.2‰) failed to reflect the seasonal variability of δ^13^C_A_Picarro_ and δ^13^C_A_model_ (Fig. 2A, E). Also, δ^13^C_pinitol_ in both 1N and 0N was over 3‰ lower than δ^13^C_A_Picarro_ and δ^13^C_A_model_.

### iWUE and environmental signals in δ^13^C of leaf carbon pools

The Spearman correlations calculated for δ^13^C of different leaf carbon pools and the series of δ^13^C_A_model_, δ^13^C_A_Picarro_, and several environmental variables, to examine aims (i)–(iii), as defined in Introduction are shown in Fig. 4. δ^13^C_starch_ and δ^13^C_pinitol_ in both 1N and 0N, as well as δ^13^C_TOM_ in 1N, did not correlate with the tested variables, and were hence excluded from Fig. 4.

Aim (i): δ^13^C_sucrose_ correlated better with δ^13^C_A_model_ and δ^13^C_A_Picarro_ compared with δ^13^C of the other tested carbon pools (Fig. 4). Aim (ii): δ^13^C_sucrose_ had better correlations with *T* and VPD than δ^13^C of the other studied carbon pools, but recorded similar PAR and RH signals as δ^13^C_WSC_ (Fig. 4). Aim (iii): among all tested carbon pools, sucrose recorded clearly the strongest iWUE signal (Fig. 4).

Overall, the strength of correlation tended to increase, when a carry-over effect was considered (Fig. 4). For δ^13^C_sucrose_, the carry-over effect was most notable, when a fraction of the previous day’s signal from the past 3–5 d was incorporated into iWUE_gas_ and the environmental variables (Fig. 4; Supplementary Fig. S4A). In addition, the correlation strength for δ^13^C_sucrose_ tended to increase, when a previous day weight of 0.7–0.8 was integrated (Fig. 4; Supplementary Fig. S4B).

### Comparing iWUE estimates

To evaluate the accuracy of intra-seasonal iWUE_iso_ derived from δ^13^C of different leaf carbon pools, we compared iWUE_iso_ with iWUE_gas_ with a carry-over effect of 4 d and a previous day weight of 0.8.

**Fig. 5.**
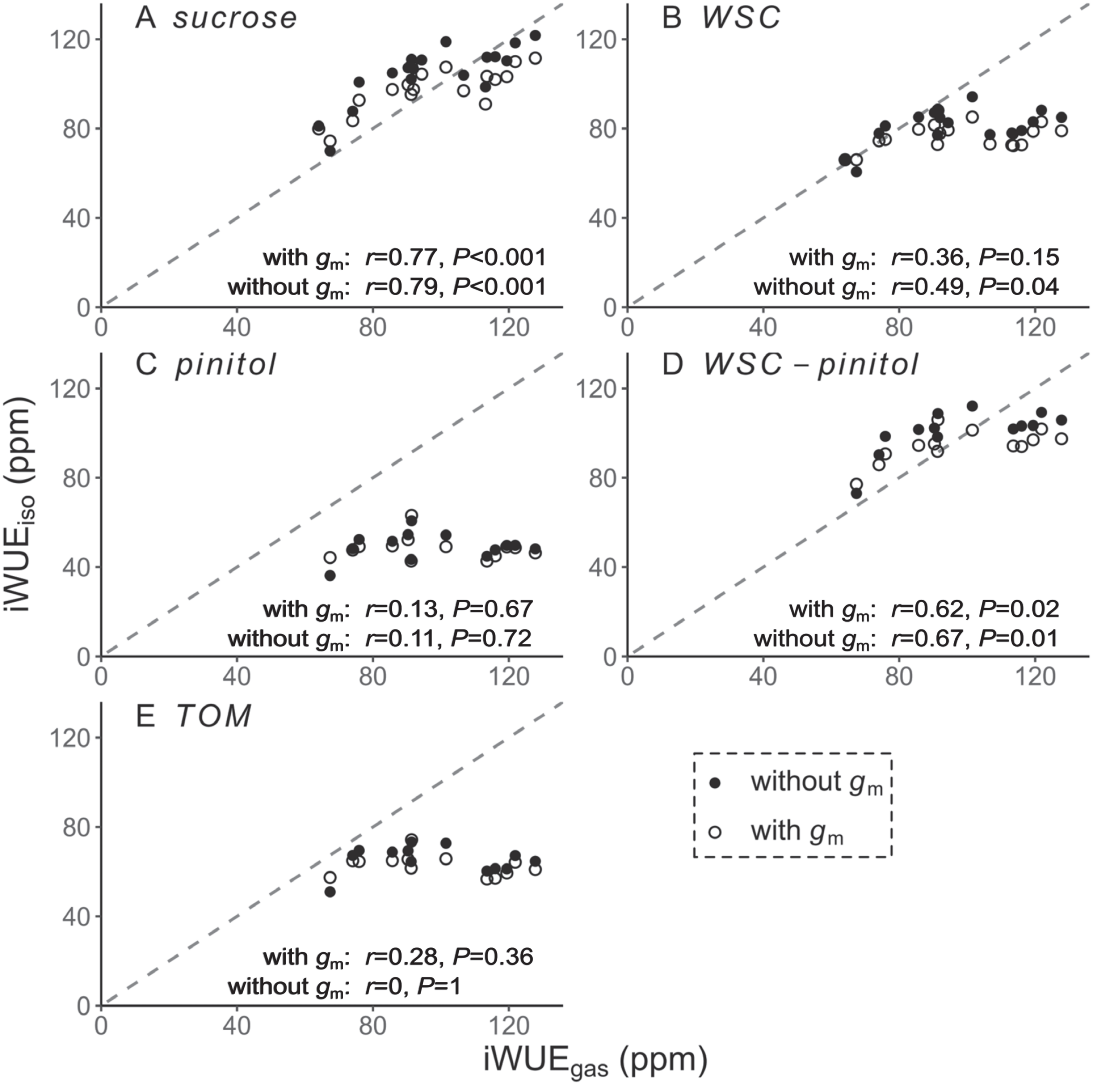
Intrinsic water use efficiency derived from leaf gas exchange (iWUE_gas_) plotted against iWUE derived from δ^13^C (iWUE_iso_) of leaf (A) sucrose, (B) pinitol, (C) water-soluble carbohydrates (WSC), (D) WSC with the deduction of pinitol contribution, and (E) total organic matter (TOM). iWUE_iso_ values were estimated by a simple model (Eq. 12) and a complex model (Eq. 11), in the former of which mesophyll conductance (*g*_m_) was not incorporated. For sucrose and WSC, iWUE_iso_ was estimated from the combined δ^13^C time series (Supplementary Fig. S3). For pinitol, TOM, and WSC with the deduction of pinitol contribution, iWUE_iso_ was estimated from the δ^13^C time series of 1-year-old need les (1N) only. Leaf gas exchange measurements were done on 1N. The gray dashed line presents *y*=*x*. Pearson’s correlation coefficient *r* and *P*-value are presented.

iWUE_iso_ estimated from δ^13^C_sucrose_ significantly correlated with iWUE_gas_, independent of whether *g*_m_ was (Pearson’s *r*=0.77, *P*<0.001, Eq. 11) or was not (Pearson’s *r*=0.79, *P*<0.001, Eq. 12) incorporated into the iWUE_iso_ estimation (Fig. 5A). Incorporating *g*_m_ improved the overall accuracy of δ^13^C_sucrose_-derived iWUE_iso_ from 104 ± 13 ppm to 98 ± 10 ppm, in comparison with iWUE_gas_ (94 ± 21 ppm). However, for the hottest period from 11 July to 2 August, δ^13^C_sucrose_-derived iWUE_iso_ was in better agreement with iWUE_gas_ (112 ± 15 ppm) without the incorporation of *g*_m_ (109 ± 10 ppm versus 100 ± 9 ppm). With and without the incorporation of *g*_m_, δ^13^C_sucrose_ overestimated iWUE_gas_ by 14 ± 4 ppm and 6 ± 5 ppm, respectively, when iWUE_gas_ was lower than 105 ppm, but underestimated iWUE_gas_ by 10 ± 5 ppm and 16 ± 6 ppm, respectively, when iWUE_gas_ was higher than 105 ppm (Fig. 5A).

iWUE_iso_ estimated from δ^13^C_WSC_ was significantly correlated with iWUE_gas_, when *g*_m_ was not considered (Pearson’s *r*=0.49, *P*=0.04, Fig. 5B), but the correlation was rather weak and the absolute values overall lower than that of iWUE_gas_ (ANOVA *P*=0.002). δ^13^C_pinitol_ produced iWUE_iso_ values that did not respond to changes in iWUE_gas_ (Fig. 5C). Considering the dampening effect on δ^13^C_WSC_ caused by isotopic invariant pinitol (–31.0 ± 0.3‰), which was a major component of WSC (40 ± 6%), we corrected δ^13^C_WSC_-based iWUE_iso_ by deducting the contribution of pinitol. By using the measured shares of pinitol and assuming a constant δ^13^C_pinitol_ (–31‰), the correlation between iWUE_iso_ and iWUE_gas_ was improved (Pearson’s *r*=0.67, *P*=0.01, Fig. 5B, D) and the absolute values became comparable. With a further assumption of a constant share of pinitol to WSC (40%), the corrected iWUE_iso_ from δ^13^C_WSC_ still showed similar variability (Pearson’s *r*=0.54, *P*=0.05) and absolute values with iWUE_gas_ when *g*_m_ was not considered. No significant positive correlations existed between iWUE_gas_ and iWUE_iso_ estimated from δ^13^C_TOM_ (Fig. 5E). Overall, iWUE_iso_ estimated from δ^13^C_WSC_ and δ^13^C_TOM_ tended to underestimate iWUE_gas_ and the degree of underestimation varied with time. For instance, δ^13^C_WSC_-based iWUE_iso_ differed from iWUE_gas_ by –34% to 7% with *g*_m_ or –38% to 3% without *g*_m_; δ^13^C_TOM_-based iWUE_iso_ deviated from iWUE_gas_ by –49% to –8% with *g*_m_ or –52% to –12% without *g*_m_.

## Discussion

In the current study, we present the first comparison of chamber-derived δ^13^C_A_Picarro_ and modeled δ^13^C_A_model_ with δ^13^C_sucrose_, δ^13^C_WSC_, δ^13^C_starch_, δ^13^C_pinitol_, and δ^13^C_TOM_ in leaves at a high-resolution intra-seasonal scale as a case study on mature Scots pine trees. Our results show that δ^13^C_sucrose_ aligned better with both δ^13^C_A_Picarro_ and δ^13^C_A_model_ (Fig. 3), in both absolute values and temporal variability, and correlated better with VPD, *T*, and iWUE_gas_ (Fig. 4) in comparison with δ^13^C of the other leaf carbon pools. As a result, iWUE_iso_ derived from δ^13^C_WSC_ or δ^13^C_TOM_ underestimated iWUE_gas_ and failed to reflect the seasonal changes in iWUE_gas_, whereas δ^13^C_sucrose_-based iWUE_iso_ was comparable to iWUE_gas_ (Fig. 5). Our results address the potential danger of misinterpreting iWUE and environmental signals derived from δ^13^C of bulk organic matter in leaves, and underline the advantage of using δ^13^C_sucrose_ to decipher photosynthetic carbon isotope discrimination and plant physiological processes.

### Validity and uncertainty of chamber-derived and modeled δ^13^C_A_

On one hand, the general alignment of δ^13^C_A_Picarro_, δ^13^C_A_model_, and δ^13^C_sucrose_ in their seasonal changes and absolute values (Fig. 3) supports the validity of δ^13^C_A_ data obtained from the online chamber-Picarro measurements and from the photosynthetic isotope discrimination model. On the other hand, it indicates that leaf δ^13^C_sucrose_ was overall not impacted by the use of reserves, e.g. starch, which had a different δ^13^C signal in respect to new assimilates. The detected low degree of use of reserves is also consistent with the finding of Rinne *et al.* (2015a) for *Larix gmelinii* in Siberia.

However, detailed comparison also revealed inconsistencies between the three δ^13^C series, specifically between 11 July and 2 August (Fig. 3), the period with the highest *T* and radiation. Five possible reasons for the inconsistencies during this period were identified. First, δ^13^C_A_model_ may have been overestimated, because a constant *g*_m_ value was used (Supplementary Fig. S5). As *g*_m_ may steeply increase with *T* (von Caemmerer and Evans, 2015; Shrestha *et al.*, 2019), underestimation of *g*_m_ under high *T* would lead to overestimation of δ^13^C_A_model_ (Eqs 4, 10). Second, net CO_2_ fluxes captured via the gas exchange system incorporate the mitochondrial respired ^13^C-enriched CO_2_ (Gessler *et al.*, 2009b), which may derive from respiratory substrates disconnected from recent photosynthates (Wingate *et al.*, 2007; Busch *et al.*, 2020). Hence, enhanced respiration rates under the high *T* together with the possible changes in δ^13^C of respiratory substrates may have decoupled δ^13^C_A_Picarro_ from δ^13^C_A_model_ and δ^13^C_sucrose_ on 19 July. Third, air temperature in the closed chamber increases more when radiation is high. If the needles sampled outside the chamber for sucrose analysis face different conditions than the needles used for gas exchange measurements, from which δ^13^C_A_Picarro_ and δ^13^C_A_model_ were derived, the δ^13^C results may differ. Fourth, whereas average δ^13^C_sucrose_ from five trees can be considered site-representative (Leavitt and Long, 1984), δ^13^C_A_Picarro_ and δ^13^C_A_model_ are based on measurements from two chambers installed on only one tree and may thus be more subject to uncertainties during the period of study. Fifth, further discrepancies between the three δ^13^C series may partly arise from the fact that δ^13^C_sucrose_ is a time-integrated signal over the past 3–5 d (Supplementary Fig. S4A), whereas δ^13^C_A_Picarro_ and δ^13^C_A_model_ are daily indexes. However, the higher δ^13^C_A_model_ values in respect to δ^13^C_A_Picarro_ and δ^13^C_sucrose_ between 11 July and 2 August are not likely caused by underestimated photorespiration effects in the model, since a higher photorespiration component would generate even higher δ^13^C_A_model_ values.

In addition, δ^13^C_sucrose_ was less negative compared with δ^13^C_A_model_ and δ^13^C_A_Picarro_ on 4 July and 7 September (Fig. 3). On 4 July, the ^13^C-enrichement in sucrose can be assigned to the fact that δ^13^C_sucrose_ integrated a previous 3–5 d signal (Supplementary Fig. S4A) with relatively high PAR conditions compared with the rainy sampling day (previous 5 d mean versus current day: 569 versus 256 µmol m^−2^ s^−1^). On 7 September, at the end of the dry period, the higher δ^13^C_sucrose_ signal may be related to use of starch reserves, which were ^13^C-enriched relative to δ^13^C_A_model_ and δ^13^C_A_Picarro_ (Fig. 3). At this time, water stress may have induced the conversion of starch to sugars for osmoregulation (Talbott and Zeiger, 1998). Indeed, a slight decrease in starch content (Supplementary Fig. S6D) and a corresponding increase in WSC content (Supplementary Fig. S6C) were observed in needles from 29 August to 7 September. During the dry period, stomatal conductance was relatively low (Supplementary Fig. S7), but δ^13^C_A_model_ and δ^13^C_A_Picarro_ were not at higher levels compared with other periods (Fig. 3). This is because low assimilation rates at this period (Supplementary Fig. S7), due to declining PAR and *T* conditions (Fig. 1A), outweighed the effect of stomatal closure, resulting in high *c*_i_/*c*_a_ value and thereby low δ^13^C_A_ values (Fig. 3).

### Blurring of iWUE and environmental signals in δ^13^C of bulk organic matter

δ^13^C_TOM_ values were consistently higher in 0N compared with 1N (Fig. 2F). In previous studies, both higher (Li *et al.*, 2007; Yan *et al.*, 2013; Song *et al.*, 2015) and lower (Brendel *et al.*, 2003) δ^13^C_TOM_ values have been reported in 0N in comparison with 1N for conifers. This difference likely arises from differences in environmental conditions during leaf growth periods, when the major components of TOM, i.e. the structural compounds as well as some non-structural compounds with long turnover rates, were formed. For instance, our sampling season had higher PAR and *T* during the leaf growth period in comparison with the previous year (Supplementary Fig. S8). Consequently, photosynthates of the study year were formed at higher photosynthetic rates, which provided carbon with higher δ^13^C values for 0N growth (Fig. 2F) (Farquhar *et al.*, 1989). In the study of Brendel *et al.* (2003), the sampling year had wetter conditions than the preceding year, which led to higher stomatal conductance and lower δ^13^C of photosynthates for 0N growth.

It is unlikely that the higher δ^13^C_TOM_ values in 0N relative to 1N are due to use of ^13^C-enriched reserves. If old reserves were used for early leaf growth, δ^13^C_TOM_ in 0N would be decoupled from δ^13^C_A_. In contrast, δ^13^C_TOM_ in 0N aligned with δ^13^C_A_model_ and δ^13^C_A_Picarro_ during the initial stages of leaf growth (Fig. 3), which indicates that 0N growth relied on recent photosynthates rather than reserves. Low dependence of leaf growth on reserves has been reported also for evergreen conifer *Pinus uncinata* Ramond (von Felten *et al.*, 2007), deciduous conifer *Larix gmelinii* (Rinne *et al.*, 2015a), as well as for a variety of deciduous and evergreen broadleaf species (Villar-Salvador *et al.*, 2015 and references therein; but see February and Higgins, 2016). Although the use of new assimilates for 0N growth shows the potential of using δ^13^C_TOM_ in expanding leaves for tracing δ^13^C_A_, the time window of this application is limited. In the current study, δ^13^C_TOM_ in 0N became decoupled from δ^13^C_A_model_ and δ^13^C_A_Picarro_ already somewhat before the full maturation of 0N (Fig. 3). This decoupling is probably due to high proportions of already formed needle matter and low contributions of new assimilates in TOM; for example, sucrose accounted for only 4% of TOM (Supplementary Fig. S6A).

Given that leaf δ^13^C_TOM_ in 0N was dampened outside the main leaf growth period and that in 1N remained invariant throughout the whole season (Fig. 2F), leaf TOM is not particularly good material for identifying seasonal changes in iWUE or environmental signals (Fig. 4). In addition, isotope fractionation during secondary metabolism (Brugnoli and Farquhar, 2000) further causes δ^13^C_TOM_ to deviate from δ^13^C_A_ (Fig. 3), which can lead to unreliable reconstructions of physiological or environmental signals (Fig. 5E). Such issues may also be present in the work of Tarin *et al.* (2020), where a clear seasonal trend in water use efficiency was observed from leaf gas exchange and eddy covariance data, but not from leaf δ^13^C_TOM_. Furthermore, attributing leaf δ^13^C_TOM_ to an integrated signal of the whole growing season should be avoided, as the signal is mostly determined during the initial growth period.

The iWUE, VPD, and *T* signals in δ^13^C_WSC_ were also dampened in the current study (Figs 4, 5B), as has been reported for mature mountain pine (Churakova *et al.*, 2018). This result is in contrast to the report by Brugnoli *et al.* (1988) that carbon isotope discrimination in WSC (‘soluble sugars’ therein) correlated strongly with *c*_i_/*c*_a_. However, in Brugnoli *et al.* (1988), plants were placed in darkness prior to sampling in order to consume all the previous day’s reserves. In that case, newly formed WSC would reflect the current-day iWUE_gas_. But depletion of carbon reserves is not the typical occurrence in most studies that have used δ^13^C_WSC_ as environmental and physiological indexes. In our study, the blurring in δ^13^C_WSC_ is mainly because of the almost invariant but distinctly lower δ^13^C values of pinitol (Fig. 2E), which contributed approximately 40% to the WSC pool (Supplementary Fig. S6B, C). As a result of blurred δ^13^C_WSC_ signal, δ^13^C_WSC_-based iWUE_iso_ values deviated from iWUE_gas_, as also observed by Tarin *et al.* (2020). However, we demonstrated that there was a good potential to correct δ^13^C_WSC_-based iWUE_iso_ by deducting the impact of pinitol on δ^13^C_WSC_ (Fig. 5D), even with a simplified assumption of constant δ^13^C (–31‰) and share (40%) of pinitol. This is because the other three main components of WSC (i.e. sucrose, glucose, and fructose) highly correlate in their δ^13^C values (Rinne *et al.*, 2015a) and δ^13^C_sucrose_ recorded iWUE_gas_ rather accurately (Fig. 5A). Since invariant pinitol δ^13^C values (Rinne *et al.*, 2015a; Churakova *et al.*, 2019) and a constant share of pinitol to WSC content (Rinne *et al.*, 2015a) at intra-seasonal scale were also observed for other tree species, this simple correction for δ^13^C_WSC_-based iWUE_iso_ may be applied in future studies in cases in which CSIA is not available.

Similar to WSC, we can expect a blurred environmental signal in δ^13^C of water-soluble organic matter, which contains in addition to WSC amino acids, organic acids, and phenolic compounds (Antonova and Stasova, 2006). The lack of an environmental signal in δ^13^C_starch_ is not surprising, considering that starch accumulates over time (Supplementary Fig. S6D) and therefore consists of carbon formed at different times. Taken together, our data on δ^13^C of different leaf carbon pools revealed that the iWUE and environmental signals were blurred or even distorted in δ^13^C of bulk organic matter, underlining a danger of misinterpreting these signals reconstructed from δ^13^C of bulk matter.

### δ^13^C of leaf sucrose is a good index for iWUE

Leaf δ^13^C_sucrose_ responded sensitively to iWUE and environmental variables (Fig. 4) but with a typical carry-over effect of 3–5 d (Supplementary Fig. S4A). The short-term carry-over effect, albeit slightly lower than the 2 d detected for leaf sucrose in *Larix decidua* (Streit *et al.*, 2012), is in line with that reported for leaf WSC in *Pinus pinaster* (1–5 d, Desalme *et al.*, 2017) and leaf sugars in *Pinus sylvestris* (2 to over 5 d, Leppä *et al.*, 2022). This carry-over effect is also in accordance with the finding of a two-compartment pool of sucrose in mesophyll cells (Kouchi and Yoneyama, 1984; Nadwodnik and Lohaus, 2008), i.e. a quick sucrose transport pool in the cytosol and a slow sucrose transport pool in the vacuole (Brauner *et al.*, 2014; Bögelein *et al.*, 2019). Our results indicate that approximately 70–80% of sucrose is stored in the slow turnover pool, as shown by the relatively high correlations between δ^13^C_sucrose_ and environmental variables, when a previous day weight of 0.7–0.8 on environmental variables was considered (Fig. 4; Supplementary Fig. S4B). This corresponds reasonably well with a previous report stating that 40–80% of leaf sucrose was stored in the vacuole for various non-tree plant species (Nadwodnik and Lohaus, 2008).

When *g*_m_ was not incorporated, iWUE_iso_ from δ^13^C_sucrose_ was overestimated by 9% in comparison with iWUE_gas_ over the growing season (Fig. 5A). This overestimation stems from the simplified assumption that *g*_m_ is infinite (Stangl *et al.*, 2019). Nevertheless, the impact of omitting *g*_m_ from the iWUE_iso_ calculation was less significant in our study than in a study of crop species (Ma *et al.*, 2021), where the omission caused an overestimation of up to 65% on iWUE_iso_. This is probably due to species-specific differences in *g*_m_ dynamics (Ubierna *et al.*, 2017). Considering that the overestimation of iWUE_iso_ when using an infinite *g*_m_ was within a reasonable range, there may be a better chance to reconstruct seasonal iWUE_iso_ in this simplified manner from Scots pine compared with species that have shown a significant impact of *g*_m_. Although including *g*_m_ did improve the overall accuracy of iWUE_iso_ estimates, as also observed by Gimeno *et al.* (2021), this improvement was not seen during the hottest period. This is because a constant *g*_m_ value was used in our iWUE_iso_ estimation, due to the current lack of understanding of *g*_m_ dynamics (Busch *et al.*, 2020), although *g*_m_ may in fact sensitively respond to *T* (von Caemmerer and Evans, 2015; Shrestha *et al.*, 2019). Our results imply that δ^13^C of sucrose would be a useful tool to examine the *g*_m_ dynamics, especially in extreme conditions, in future studies.

Although there was a significant linear correlation between δ^13^C_sucrose_-derived iWUE_iso_ and iWUE_gas_, there was a shift in their relationship (Fig. 5A). When iWUE_gas_ was below 105 ppm, iWUE_iso_ was higher than iWUE_gas_ (Fig. 5A). This can be explained by higher *c*_a_ in the ambient air than inside the chambers, considering that this difference in *c*_a_ was up to 30 ppm in our study. According to the reported rate of increase in iWUE with respect to *c*_a_ (0.28 ppm ppm^−1^ in Adams *et al.*, 2020), the 30 ppm difference in *c*_a_ would cause an iWUE offset of 8 ppm, in line with the observed difference between iWUE_gas_ and iWUE_iso_ of 14 ± 4 ppm and 6 ± 5 ppm with and without *g*_m_ constraints, respectively. However, when iWUE is high, the sensitivity of iWUE to increasing *c*_a_ may be reduced according to Waterhouse *et al.* (2004). Instead, the higher temperatures inside the chambers than in the ambient air may result in higher iWUE_gas_ than iWUE_iso_. On sunny days, temperature inside the chambers was approximately 2 °C higher than ambient temperature, which led to higher *e*_s_ (Eq. 2), lower *g*_s_ (Eq. 9) and, consequently, higher iWUE_gas_ (Eq. 8) by approximately 12 ppm. This temperature effect corresponds to the observed offsets between iWUE_gas_ and iWUE_iso_ of 10 ± 5 ppm and 16 ± 6 ppm for limited and infinite *g*_m_ settings, respectively (Fig. 5A). Furthermore, the iWUE offset may be superimposed by seasonal changes in the rate of sucrose export, which may prefer the export of ^13^C-enriched sucrose and thereby lower δ^13^C of the remaining leaf sucrose (Bögelein *et al.*, 2019). In other words, a higher rate of sucrose export may lower leaf δ^13^C_sucrose_ to a larger extent. Indeed, all days of iWUE_gas_ over 105 ppm, except 16 August, occurred right before needle growth (18 May to 25 May) and in the period with maximum tracheid maturation rate (14 July to 13 August) (Tang *et al.*, 2022), when high growth demands may have triggered high sucrose export rate and lowered leaf δ^13^C_sucrose_ and iWUE_iso_.

Since sucrose is the dominant transport sugar (Rennie and Turgeon, 2009; Julius *et al.*, 2017) for structural growth, the δ^13^C signal of leaf sucrose and therefore the leaf-level iWUE signal is laid down in tree rings. However, because the δ^13^C signal of sucrose may be modified along the leaf-to-phloem pathway (Gessler *et al.*, 2009a; Bögelein *et al.*, 2019), due to, for example, use of reserves (Helle and Schleser, 2004) or vertical mixing of assimilates (Bögelein *et al.*, 2019), the archived iWUE signal in tree ring δ^13^C may be biased to some extent. Future studies that concurrently trace intra-seasonal δ^13^C signals in leaf and phloem sucrose and in tree rings will help to quantify the changes from leaf-level iWUE to tree-level iWUE. This cannot only provide in-depth understanding about the reasons behind an offset between gas exchange-derived leaf-level iWUE and tree ring δ^13^C-derived tree-level iWUE, but also give confidence in reconstructing intra-seasonal iWUE estimates via intra-annual tree ring δ^13^C analysis (Rinne *et al.*, 2015b; Soudant *et al.*, 2016).

### Conclusion

This work has presented the first high-resolution intra-seasonal comparison between chamber-derived δ^13^C_A_Picarro_, modeled δ^13^C_A_model_, and δ^13^C of different leaf carbon pools, some of which have been used to reconstruct iWUE. We observed that both δ^13^C_A_Picarro_ and δ^13^C_A_model_ generally aligned in absolute values and seasonal changes with δ^13^C_sucrose_, but not with δ^13^C of the other leaf carbon pools. This confirms the validity of the δ^13^C_A_Picarro_ and δ^13^C_A_model_ data and at the same time an insignificant reserve signal in leaf sucrose. The short-term discrepancies between δ^13^C_A_Picarro_, δ^13^C_A_model_, and δ^13^C_sucrose_ reflected the dynamics in *g*_m_, mitochondrial respiration and chamber performance under high temperature conditions, implying the potential of using δ^13^C_sucrose_ to decipher the *g*_m_ dynamics. Further, we outlined the ideal environmental period that δ^13^C of each carbon pool has the ability to capture. δ^13^C_sucrose_ integrated the environmental conditions that prevailed during the period from 3 to 5 d, while δ^13^C_TOM_ best integrated the early growing season environmental conditions. δ^13^C_WSC_ was less ideal than δ^13^C_sucrose_ for capturing intra-seasonal changes in VPD, *T*, and iWUE because the high proportion of isotopically invariant pinitol in WSC dampens its δ^13^C signal. Also, we found that δ^13^C_sucrose_, but not δ^13^C of the other carbon pools, could reconstruct intra-seasonal leaf-level iWUE in a highly accurate manner. These findings invalidate the assumption of earlier studies that δ^13^C_A_ and leaf-level iWUE is clearly documented in δ^13^C of leaf bulk organic matter (e.g. WSC and TOM) at intra-seasonal scale, and thus weakens the conclusions of those studies which seek to follow environment- and physiology-driven δ^13^C signals within trees. Thereby, we address the need to carefully examine and interpret the environmental and physiological signals in δ^13^C of bulk matter and suggest the use of CSIA for an in-depth understanding of photosynthetic isotope discrimination and physiological processes.

## Supplementary data

The following supplementary data are available at *JXB* online.

Fig. S1. Shoot gas exchange chambers used in this study.

Fig. S2. Impact of light-inhibited mitochondrial respiration on modeled δ^13^C of assimilates.

Fig. S3. Combination of time-series δ^13^C data in current-year and 1-year-old needles.

Fig. S4. Occurrence of the highest correlations between environmental and physiological variables and δ^13^C of sucrose when carry-over effect is considered.

Fig. S5. Impact of mesophyll conductance on modeled δ^13^C of assimilates.

Fig. S6. Concentrations of different leaf carbohydrates of Scots pine in Hyytiälä during the growing season of 2018.

Fig. S7. Stomatal conductance and assimilation rate of Scots pine in Hyytiälä during the growing season of 2018.

Fig. S8. Comparison of photosynthetically active radiation and air temperature in Hyytiälä between 2017 and 2018.

Table S1. Results of the mixed-effects models selected for testing the difference in δ^13^C of assimilates between estimation methods.

Table S2. Results of the mixed-effects models selected for testing the δ^13^C difference between needle generations.

Table S3. Results of the mixed-effects models selected for testing the temporal trends in δ^13^C series.

erac413_suppl_Supplementary_Material

## Data Availability

The data supporting the findings of this study are available from the corresponding author (YT) upon request.

## Abbreviations

0N: current-year needles
1N: 1-year-old needles
CSIA: compound-specific isotope analysis
δ^13^C: stable carbon isotope composition
δ^13^C_A_: δ^13^C of new assimilates
δ^13^C_A_model_: δ^13^C of assimilates estimated by the photosynthetic isotope discrimination model
δ^13^C_A_Picarro_: δ^13^C of assimilates estimated by the chamber-Picarro system
δ^13^C_pinitol_: δ^13^C of pinitol
δ^13^C_starch_: δ^13^C of starch
δ^13^C_sucrose_: δ^13^C of sucrose
δ^13^C_TOM_: δ^13^C of total organic matter
δ^13^C_WSC_: δ^13^C of water-soluble carbohydrates
*g*_m_: mesophyll conductance
iWUE: intrinsic water use efficiency
iWUE_gas_: intrinsic water use efficiency estimated from gas exchange data
iWUE_iso_: intrinsic water use efficiency estimated from δ^13^C of leaf carbon pools
PAR: photosynthetically active radiation
RH: relative humidity
*T*: air temperature
TOM: total organic matter
VPD: vapor pressure deficit
WSC: water-soluble carbohydrates
