# Supplementary material for: Estimating intra-seasonal photosynthetic discrimination and water use efficiency using δ13C of leaf sucrose in Scots pine
Source: J Exp Bot. 2022 Oct 18;74(1):321–35. doi: 10.1093/jxb/erac413 (PMC9786842; doi:10.1093/jxb/erac413)
Supplement: erac413_suppl_Supplementary_Material [file erac413_suppl_supplementary_material.pdf]

Journal of Experimental Botany Supplementary Data

Article title: Estimating the intra-seasonal photosynthetic discrimination and water use efficiency using $\delta^{13}C$ of leaf sucrose in Scots pine

Authors: Yu Tang, Pauliina Schiestl-Aalto, Marco M. Lehmann, Matthias Saurer, Elina Sahlstedt, Pasi Kolari, Kersti Leppä, Jaana Bäck, Katja T. Rinne-Garmston

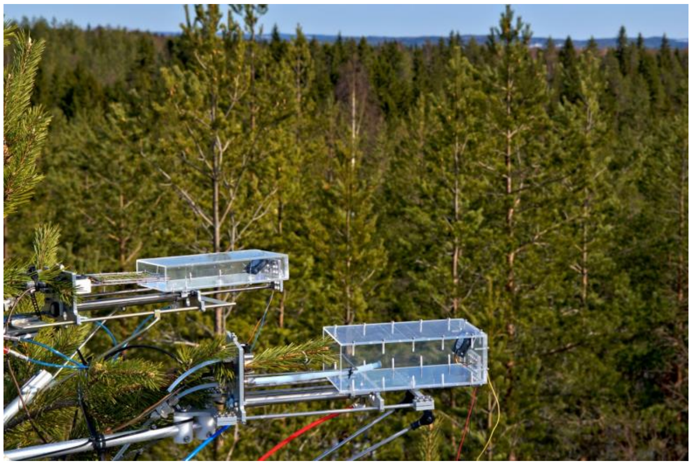

Fig. S1. Shoot gas exchange chambers used in this study (credit to Juho Aalto).

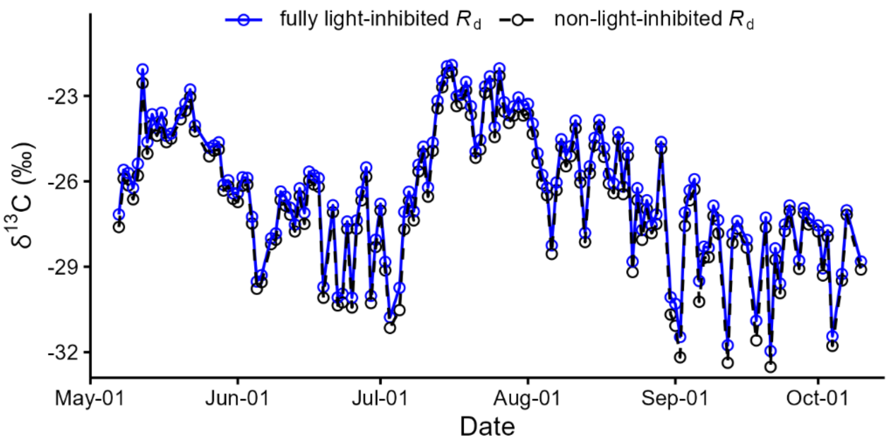

Fig. S2. Impact of light-inhibited mitochondrial respiration on modeled δ^{13}C of assimilates. δ^{13}C of assimilates was estimated by the photosynthetic isotope discrimination model with fully light-inhibited or non-light-inhibited mitochondrial respiration (R_d).

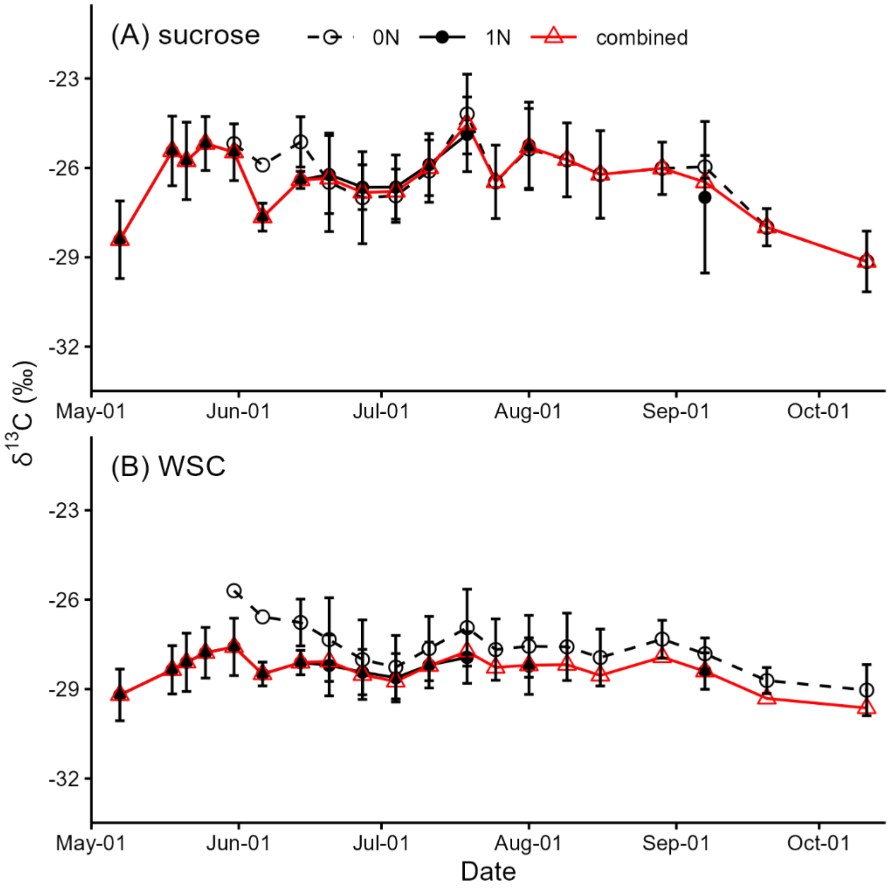

Fig. S3. Combination of time-series δ^{13}C data in current-year and one-year-old needles. (A) sucrose and (B) water-soluble carbohydrates (WSC). 0N is current-year needles, and 1N is one-year-old needles.

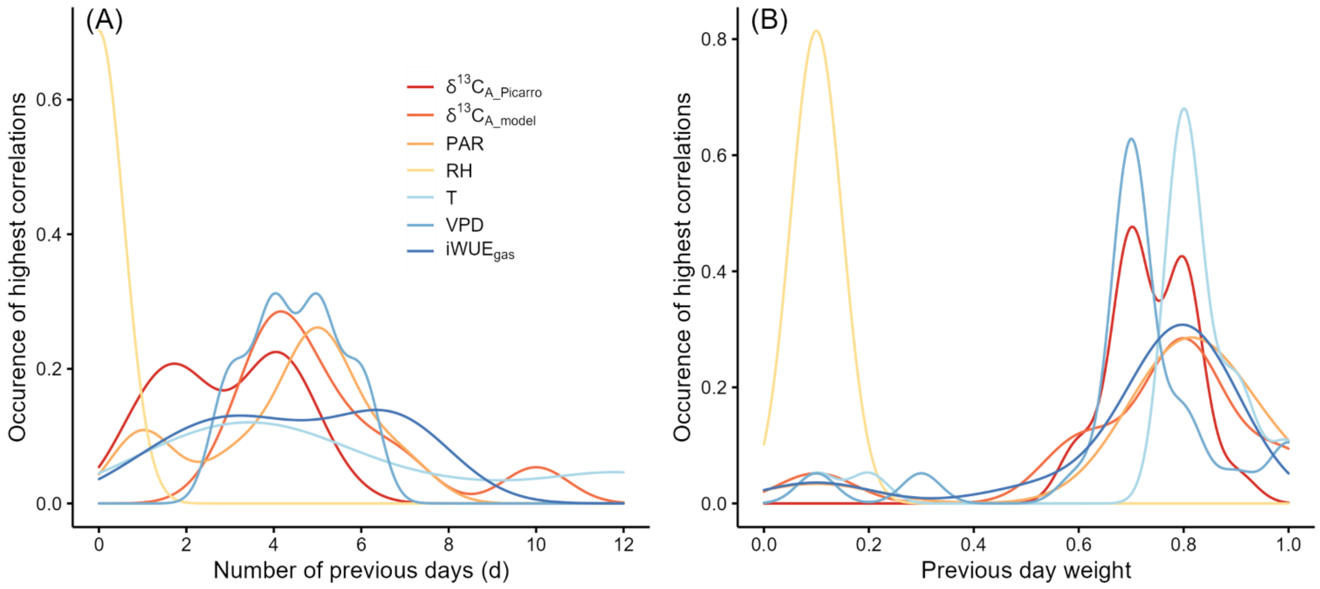

Fig. S4. Occurrence of the highest correlations between environmental and physiological variables and δ^{13}C of sucrose when carry-over effect is considered. (A) number of previous days, (B) previous day weight. Combined δ^{13}C time series of sucrose from current-year needles and one-year-old needles were analyzed. Variables analyzed include δ^{13}C of assimilates estimated from the chamber-Picarro system (δ$^{13}C_{A_Picarro}$), δ^{13}C of assimilates estimated from the photosynthetic isotope discrimination model (δ$^{13}C_{A_model}$), photosynthetically active radiation (PAR), relative humidity (RH), air temperature (T), vapor deficit pressure (VPD) and intrinsic water use efficiency estimated from gas exchange data ($iWUE_{gas}$).

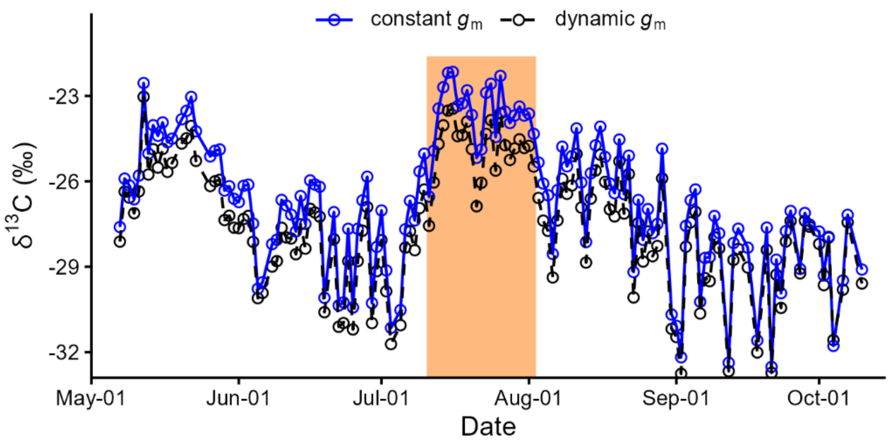

Fig. S5. Impact of mesophyll conductance on modeled $\delta^{13}C$ of assimilates. $\delta^{13}C$ of assimilates estimated by the photosynthetic isotope discrimination model with constant or dynamic mesophyll conductance (g_m) values. A constant g_m value of 0.127 mol m^{-2} s^{-1} (Stangl *et al.*, 2019) and dynamic g_m values which increase with temperature (Sun *et al.*, 2014; Schiestl-Aalto *et al.*, 2021) were used here. The period between July 11 and August 2, which had the highest temperature during the growing season, is shaded in orange.

References:

Schiestl-Aalto P, Stangl ZR, Tarvainen L, Wallin G, Marshall J, Mäkelä A. 2021. Linking canopy-scale mesophyll conductance and phloem sugar $\delta^{13}C$ using empirical and modelling approaches. New Phytologist **229**, 3141–3155.

Stangl ZR, Tarvainen L, Wallin G, Ubierna N, Räntfors M, Marshall JD. 2019. Diurnal variation in mesophyll conductance and its influence on modelled water-use efficiency in a mature boreal Pinus sylvestris stand. Photosynthesis Research **141**, 53–63.

Sun Y, Gu L, Dickinson RE, Norby RJ, Pallardy SG, Hoffman FM. 2014. Impact of mesophyll diffusion on estimated global land CO_2 fertilization. Proceedings of the National Academy of Sciences **111**, 15774–15779.

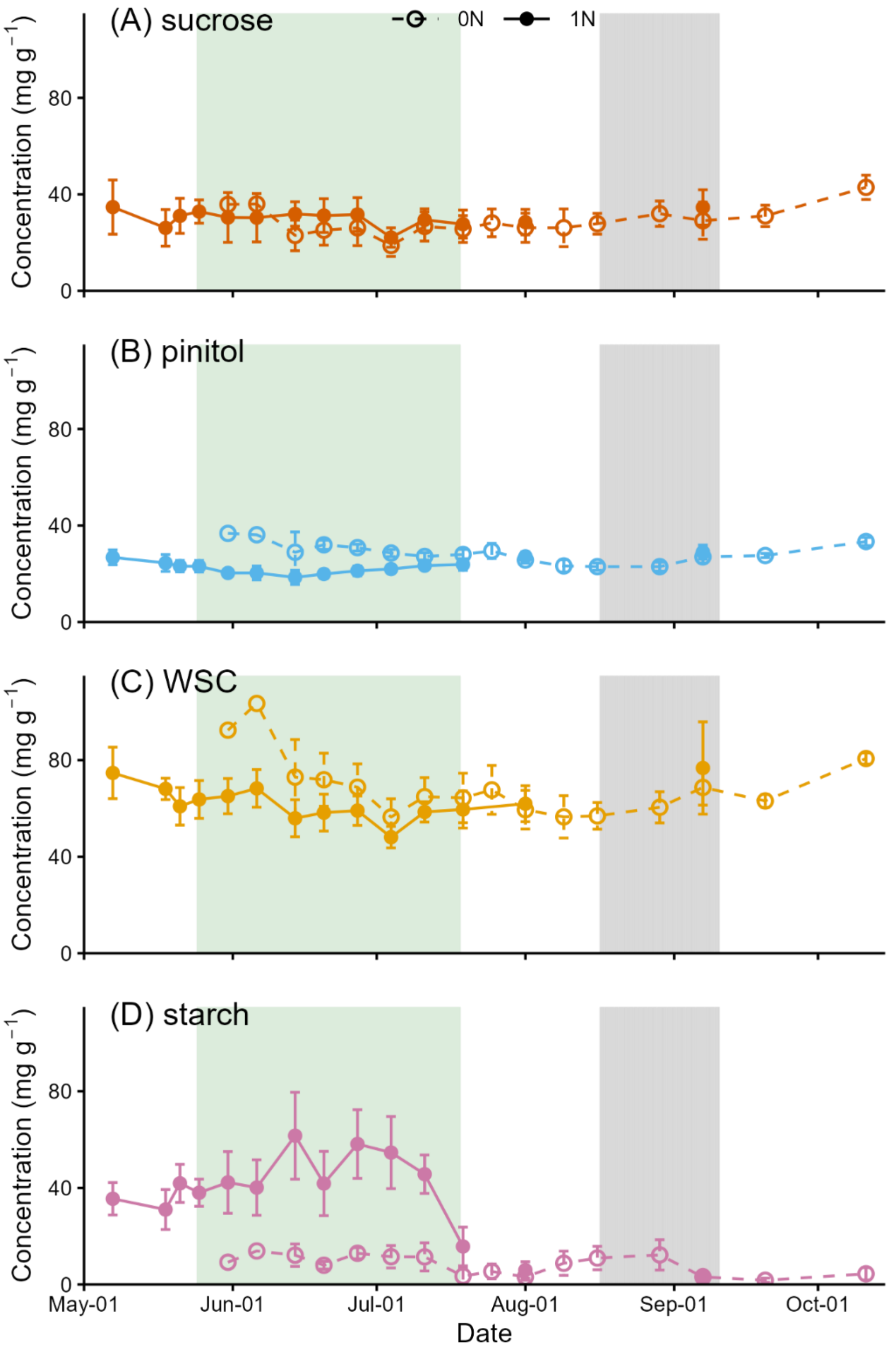

Fig. S6. Concentrations of different leaf carbohydrates of Scots pine in Hyytiälä during the growing season of 2018. (A) sucrose, (B) pinitol, (C) water-soluble carbohydrates (WSC), (D) starch. 0N is current-year needles, and 1N is one-year-old needles. The needle growth period is shaded in green, and the dry period in gray. Error bars represent SD of five trees.

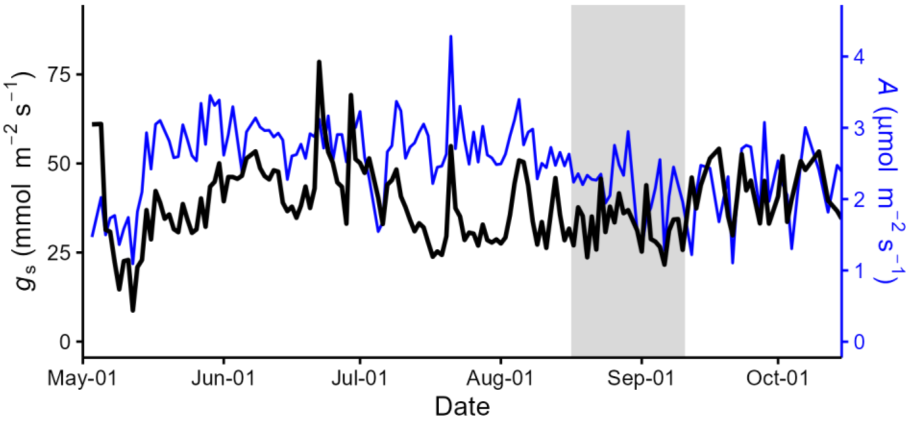

Fig. S7. Stomatal conductance and assimilation rate of Scots pine in Hyytiälä during the growing season of 2018. g_s (black line) is stomatal conductance, and A (blue line) is assimilation rate. The dry period is shaded in gray.

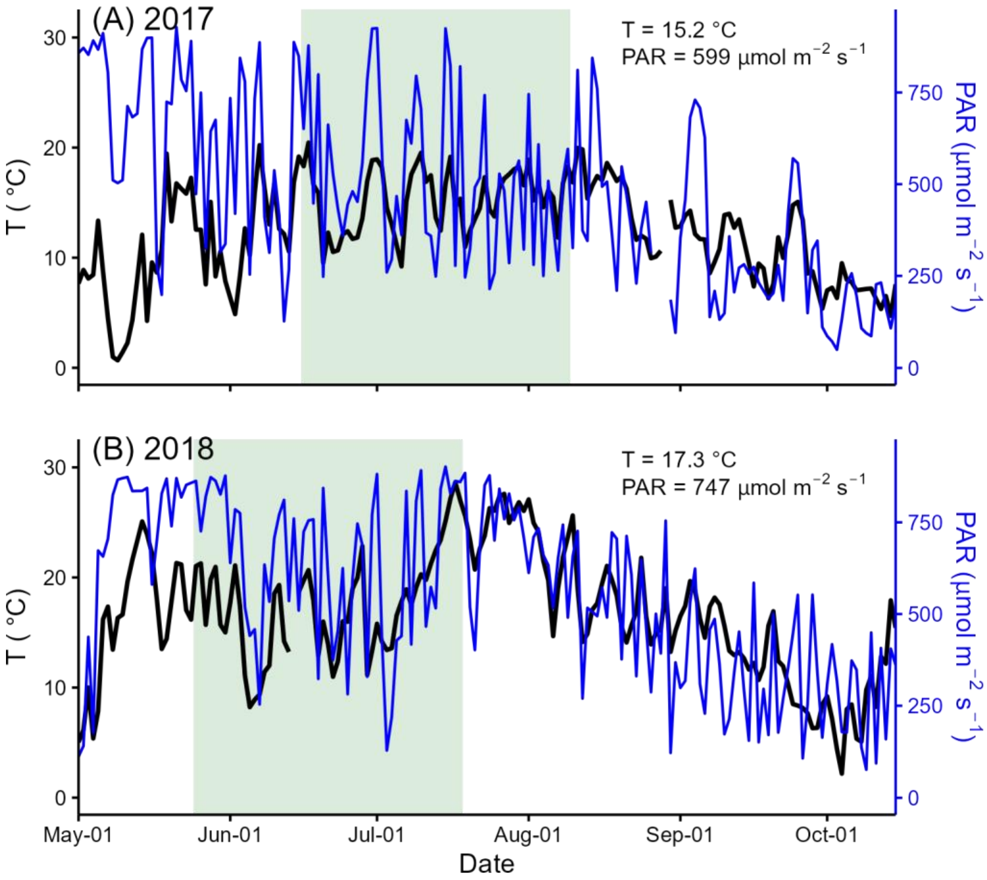

Fig. S8. Comparison of photosynthetically active radiation and air temperature in Hyytiälä between 2017 and 2018. PAR is photosynthetically active radiation, and T is air temperature. The needle growth period is shaded in green. Means of T and PAR for the needle growth period were presented.

Table S1. Results of the mixed-effects models selected for testing the difference in $\delta^{13}C$ of assimilates ($\delta^{13}C_A$) between estimation methods, i.e., via chamber-Picarro system or isotope discrimination model. Random effect of the mixed-effects models is chamber identifier (1,2). DOY is day of year. The results show that there were no significant differences ($P = 0.467$) between $\delta^{13}C_A$ estimated from the chamber-Picarro system ($\delta^{13}C_{A\_Picarro}$) and $\delta^{13}C_A$ estimated from the photosynthetic isotope discrimination model ($\delta^{13}C_{A\_model}$) for the whole growing season. However, $\delta^{13}C_{A\_Picarro}$ significantly ($P < 0.0001$) differed from $\delta^{13}C_{A\_model}$ for the period from July 11 to August 2.

Response variable	$\delta^{13}C_A$	$\delta^{13}C_A$
period	whole growing season	July 11 to August 2
P-value for		
DOY	<0.0001	0.423
method	0.467	<0.0001
DOY:method	ns	ns
Model R^2	0.19	0.73

Table S2. Results of the mixed-effects models selected for testing the $\delta^{13}C$ difference between needle generations, i.e., one-year-old needles (1N) and current-year needles (0N). Random effect of the mixed-effect models is tree identifier (1,2,3,4,5). $\delta^{13}C$ of 1N and 0N analyzed from seven sampling days were used, i.e., after the first three sampling days of 0N. DOY is day of year. TOM is total organic matter. WSC is water-soluble carbohydrates. Due to a consistent offset in $\delta^{13}C_{WSC}$ (0.6‰) between 1N and 0N (Supplementary Fig. S3B), we tested also whether the $\delta^{13}C_{WSC}$ difference between needle generations was significant after subtracting this offset from 0N data. $\delta^{13}C_{WSC}$* represents that $\delta^{13}C_{WSC}$ in 0N was corrected by a constant offset of –0.6‰.

Response variable	$\delta^{13}C_{sucrose}$	$\delta^{13}C_{WSC}$	$\delta^{13}C_{WSC}$*	$\delta^{13}C_{pinitol}$	$\delta^{13}C_{starch}$	$\delta^{13}C_{TOM}$
P-value for						
DOY	0.939	0.948	0.948	0.128	0.0003	0.116
Generation	0.410	<0.0001	0.771	<0.0001	0.0017	<0.0001
DOY:generation	ns	ns	ns	ns	0.0007	ns
Model R^2	0.46	0.80	0.78	0.83	0.71	0.92

Table S3. Results of the mixed-effects models selected for testing the temporal trends in $\delta^{13}C$ series. $\delta^{13}C_{A\_Picarro}$ is $\delta^{13}C$ of assimilates estimated by the chamber-Picarro system, $\delta^{13}C_{A\_model}$ is $\delta^{13}C$ of assimilates estimated by the photosynthetic isotope discrimination model, $\delta^{13}C_{sucrose}$ is $\delta^{13}C$ of sucrose, $\delta^{13}C_{TOM}$ is $\delta^{13}C$ of total organic matter, and DOY is day of year.

Response variable	$\delta^{13}C_{A\_Picarro}$	$\delta^{13}C_{A\_model}$	$\delta^{13}C_{sucrose}$	$\delta^{13}C_{A\_Picarro}$	$\delta^{13}C_{A\_model}$	$\delta^{13}C_{TOM}$
Random effect	chamber identifier (1,2)	chamber identifier (1,2)	tree identifier (1,2,3,4,5)	chamber identifier (1,2)	chamber identifier (1,2)	tree identifier (1,2,3,4,5)
period	August 1 to October 15	August 1 to October 15	August 1 to October 15	May 20 to June 30	May 20 to June 30	May 20 to June 30
P-value for DOY	0.013	<0.0001	0.024	0.004	<0.0001	0.005
Slope for DOY	–0.03	–0.06	–0.03	–0.06	–0.11	–0.05
Model R^2	0.05	0.59	0.53	0.15	0.71	0.76
